# Exploring the roles of paradoxical tensions, paradoxical thinking, and team psychological capital on the creativity of engineering university students

**DOI:** 10.1186/s40359-025-02427-3

**Published:** 2025-02-12

**Authors:** Huifen Guo, Zhen Zhou, Fengqi Ma

**Affiliations:** 1 School of Education, Guangzhou University, Guangzhou, Guangdong China; 2 School of Mechanical and Electrical Engineering, Guangzhou University, Guangzhou, Guangdong China

**Keywords:** Paradoxical tensions, Paradoxical thinking, Creativity, Team psychological capital

## Abstract

**Background:**

The multifaceted challenges encountered by engineering university students generate paradoxical tensions, which serve as catalysts for fostering creativity. Engaging in paradoxical thinking during academic pursuits enhances students’ ability to solve complex engineering problems. Despite this, the intricate interconnections among paradoxical tensions, paradoxical thinking, and the creativity of engineering university students remain ambiguous.

**Methods:**

This study aimed to explore the gap by surveying 1,410 engineering students in China, examining how paradoxical thinking mediates the relationship between paradoxical tensions and creativity. Additionally, it investigated the moderating impact of team psychological capital on the associations between paradoxical tensions and both paradoxical thinking and creativity. SPSS 24.0 was initially used to convert the cleaned data into a “.csv” format, and Smart PLS (v.4.0.9.5) was then employed to assess the model.

**Results:**

The findings reveal a positive influence of paradoxical tensions on creativity and thinking. Notably, paradoxical thinking emerges as a significant contributor to enhancing the creativity of engineering university students. Furthermore, the findings show that paradoxical tensions enhance creativity by influencing paradoxical thinking. While team psychological capital emerged as a significant factor in moderating the link between paradoxical tension and creativity, its role in moderating the association between paradoxical tension and paradoxical thinking was not statistically significant.

**Conclusions:**

This study revealed how paradoxical tensions among engineering university students influence creativity through paradoxical thinking moderated by team psychological capital. The findings provide new insights for researchers to understand paradoxical tensions, paradoxical thinking, team psychological capital, and the underlying psychological mechanism for engineering university students’ creativity better, and they have practical implications for education administrators.

**Supplementary Information:**

The online version contains supplementary material available at 10.1186/s40359-025-02427-3.

## Introduction

Creativity is an indispensable trait for engineering students, exerting a profound influence on competitiveness and the nation’s economic trajectory [1]. In industries driven by innovation, engineers endowed with creative prowess are invaluable, contributing to developing cutting-edge technologies and strengthening key sectors. Creatively inclined engineers offer immeasurable societal benefits, thinking beyond conventional boundaries to enhance community life and address global challenges [2]. Meanwhile, exposing engineering students to paradoxes and dilemmas is advantageous as it highlights the formation of social perceptions within the engineering domain [3]. Encouraging engineering students to interpret contradictions and paradoxes is essential for fostering a nuanced understanding of complex problems, which are inherently part of engineering education [4].

What is Creativity in Engineering University Students? Scholars have provided different interpretations of the concept of creativity among engineering students. Bremer et al. (2010) suggested that exposing students to innovation and an inventive culture, facilitated by the articulation of complex and challenging problems, is instrumental in eliciting heightened levels of engagement and creative problem-solving prowess [5]. Byrge and Hansen (2009) defined creativity as the boundless application of knowledge within the realms of thought and action [6]. Charyton et al. (2011) delineated engineering creativity as a construct encompassing both the dimensions of novelty or originality, which pertains to the generation of new ideas, and usefulness, which relates to the practical application of those ideas [7]. Cropley and Cropley (2000) characterize creative engineers as individuals who are motivated to pursue uniqueness, possess the capacity for unconventional thinking, embrace the non-traditional, and actively seek out the unforeseen implications of their ideas [8]. Hey et al. (2008) proposed that creativity, often drawing analogies from nature or existing problems, frequently aids engineers in devising innovative solutions [9]. León-Rovira et al. (2008) contended that creativity is the capacity to apply knowledge in a manner that addresses problems and yields original works that are recognized and valued by society [10]. Shoop and Ressler (2011) proposed that students must possess the creativity necessary to conceive innovative solutions to the world’s challenges [11]. Silva et al. (2009) asserted that creativity within an engineering context entails the application of numerical knowledge, hardware expertise, and technological acumen to devise effective and practical engineering solutions that surpass existing ones [12]. From these definitions, it is evident that the creativity of engineering students is emphasized in two distinct aspects. Firstly, students are required to possess the ability to creatively solve engineering problems, meaning they can innovatively address real-world issues as needed. Secondly, students should be able to produce novel, effective, and useful outcomes, which may include tangible items, ideas, plans, systems, products, or processes.

One major factor influencing the creativity of engineering students is paradoxical tensions [13, 14]. Paradoxical tensions involve opposing, conflicting, or competitive elements [15, 16]. Instead of being straightforward either/or choices, these tensions are intricately connected via their inherent links. Importantly, paradoxical tensions can mutually reinforce each other, indicating a complex interplay [16, 17]. Within the extant corpus of literature about educational dynamics, scholars have uniformly discerned that tensions, which manifest as divergent viewpoints [18], competing demands, and incongruent practices, are positively correlated with elevated anxiety and stress levels [15]. These psychological states, paradoxically, also serve as catalysts for innovation, creativity, and transformative change [19]. In the scholarly discourse on innovation and creativity, tensions such as the dichotomy between the novelty and usefulness of ideas and products, the balance between exploration and exploitation, and the interplay between cooperation and competition are well-documented phenomena [20]. These tensions are integral to the creative process and are often seen as the driving forces behind the generation of creative solutions. When students have more tension, they tend to be exposed to more competing ideas simultaneously. From the information processing perspective, students are motivated to process the divergent perspectives cognitively. In this case, they are more likely to experience more tensions that are indispensable for creativity. In educational settings, increased tension among students is often associated with their concurrent encounter with a variety of competing conceptual frameworks [21]. Through the lens of cognitive information processing, such tension acts as a motivational force, prompting students to engage in the comprehensive cognitive assimilation of these contrasting viewpoints. This engagement is pivotal, as it predisposes them to confront the tensions that are inherently crucial for the cultivation of creativity, thereby facilitating the emergence of novel and useful solutions [22]. Therefore, it is crucial to explore the relationship between paradoxical tensions and student creativity. However, the extant literature presents divergent results concerning the effects of paradoxical tensions on creativity [23, 24]. Studies have indicated that paradoxical tensions can exert both positive [23, 25] and negative influences on creativity [26].

Another significant factor influencing creativity in engineering is paradoxical thinking [27]. Paradoxical thinking serves as a cognitive mechanism that empowers collegiate individuals to reassess prevailing perspectives on complex issues critically [28]. This cognitive approach facilitates an active embracement and acceptance of tensions by mentally aligning contradictory yet interconnected elements [29–31]. This cognitive modality profoundly influences two pivotal dimensions of creativity: creative thinking skills [32] and problem-identifying ability [33]. Paradoxical thinking instigates a “both/and” cognitive paradigm among university students, transcending the binary “either/or” construct, thereby enabling the reconciliation of antagonistic elements or issues and the discovery of creative solutions [15, 34]. The ability to accept and integrate multiple contradictory elements or issues is fundamental to creativity [35]. Paradoxical thinking endows university students with the ability to actively embrace and engage with tensions and interrelated, yet contradictory, issues, eschewing the urge to eradicate them [18]. When solving engineering problems with a creative mindset, students who adopt a paradoxical thinking framework demonstrate an ability to synthesize relevant elements or issues, leading to the emergence of novel concepts. This approach not only enriches the exploration of problems but also enhances the formation of diverse connections among various elements, which in turn, stimulates the generation of a broader array of original solutions. Nevertheless, the research has fallen short in thoroughly examining the nexus between paradoxical tension, paradoxical thinking, and creativity [23], with particular emphasis on the mediating role of paradoxical thinking in the relationship between paradoxical tension and creativity [27].

Team psychological capital plays a significant role in enhancing creativity among engineering students [36]. This importance is underscored by the necessity of cultivating a supportive and resilient environment [37]. Such an environment is crucial for the flourishing of creative ideas, as it fosters the conditions necessary for innovation and problem-solving to thrive [38]. Luthans et al. (2006) [39] articulate psychological capital as the positive psychological developmental state of an individual. This state is characterized by the possession of confidence to confront and invest the necessary effort in excelling at challenging tasks. It involves persisting toward goals and, when necessary, adjusting paths to achieve them. Furthermore, it encompasses maintaining resilience when confronted with problems and adversity, rebounding, and even surpassing them to achieve success. Finally, it involves maintaining a positive outlook on achieving success in both the current and future contexts. Psychological capital, similar to a state-like variable, is quantifiable, deployable, and subject to cultivation through external interventions like on-the-job training, positive feedback, or group support [40]. Despite this, there is a dearth of research examining the relationship between team psychological capital and the triad of paradoxical thinking, paradoxical tension, and creativity.

## Research gap

The identified research gap concerns the ambiguous influence of paradoxical tensions on creativity, as indicated by existing literature [27], which highlights both positive and negative outcomes. In the context of engineering education, particularly among engineering students, it is crucial to investigate whether paradoxical tensions have a positive or negative effect on their creativity. Moreover, the mediating role of paradoxical thinking in the relationship between paradoxical tensions and creativity is an area that requires further exploration. There is a scarcity of research examining the impact of team psychological capital on the interplay between paradoxical tensions and paradoxical thinking, as well as its influence on the creativity of engineering students.

## Research objectives

This study aims to construct a moderated mediation model to explore the factors influencing the creativity of engineering university students. Specifically, the model will investigate the mediating role of paradoxical thinking in the relationship between paradoxical tensions and the creativity of engineering students, as well as the moderating effect of team psychological capital on the relationship between paradoxical tensions and paradoxical thinking, and between paradoxical tensions and the creativity of engineering students.

Based on the aforementioned analysis, the research questions posed are as follows:

1. What is the impact of paradoxical tensions and paradoxical thinking on the creativity of engineering students?
2. What role does paradoxical thinking play in the correlation between paradoxical tensions and creativity in engineering students?
3. What role does team psychological capital play in positively or negatively moderating the influence between paradoxical tensions and paradoxical thinking, and between paradoxical tensions and creativity?

## Research framework and hypotheses

### Paradoxical tension and paradoxical thinking

Paradoxical tensions impact the mental sense-making processes of university students [41–42, 34]. While these tensions may evoke unease and doubt among students [43], potentially leading to an aggressive approach, they can also motivate students to cognitively acknowledge the persistent existence of paradoxical tensions and adopt a receptive stance toward opposing elements or issues. This fosters the development of a cognitive framework and thinking style characterized by paradox. Nevertheless, individual thinking styles are shaped and embedded within the broader cultural context [44]. Due to prolonged exposure to traditional Chinese culture, Chinese university students are inclined to be significantly impacted by Zhong-Yong thinking^1^ and the cognitive framework of Yin-Yang balancing^2^ [45–48]. Smith et al. [49] observed that individuals in China are more prone to adopting a paradoxical frame and thinking style when facing paradoxical situations than individuals in the United States. Prashantham and Eranova [50] noted that individuals from the East, such as those from China, more readily embrace a paradoxical frame and welcome tensions than individuals from the West. When confronting conflicting yet interconnected challenges in engineering projects, university students tend to rely on their previous frames of reference to manage and resolve issues due to cognitive path dependency [51].

However, the shifts in external environmental conditions may render former frames of reference obsolete, necessitating the reevaluation of paradoxical tensions through the cultivation of new frames of reference [52]. In situations where existing cognitive frameworks fail to align with environmental changes, experiencing tensions could act as a catalyst for students to learn through paradox, recognizing, transcending, and addressing contradictions [53]. Consequently, engineering university students can perceive paradoxical tensions as educational instruments [54], enhancing their ability to progressively comprehend and handle intricate tensions through the learning process [55]. This enhanced capacity to navigate paradoxical tensions contributes to further establishing paradoxical thinking among students, fostering a mindset that embraces and effectively manages conflicting elements. As a result, the subsequent hypothesis was postulated:

#### H1

Paradoxical tensions exert a significant influence on paradoxical thinking.

### Paradoxical thinking and creativity

Paradoxical thinking empowers individuals to proactively embrace tensions and welcome contradictory yet interconnected issues, as opposed to seeking their elimination [31]. The integration of two or more conflicting elements or matters is intrinsic to creativity [35]. Studies indicate that organizations and teams are progressively adopting frames characterized by a paradox in response to paradoxical situations, thereby influencing team and organizational performance [22, 26, 29]. The capacity of paradoxical thinking to stimulate university students’ “both/and” cognition, as opposed to “either/or” thinking, empowers them to connect opposing elements or issues and uncover new solutions [56]. This cognitive shift also fosters the development of creative thinking skills [57] and the capacity to identify problems [58]. Paradoxical cognition diminishes the inertial risk associated with established cognitive frames, allowing students to be receptive to alternative information [59] and consequently enhancing creativity [60]. Numerous studies have underscored the favorable impact of “both/and” thinking [14, 61]. When undertaking tasks that demand creativity or addressing problems innovatively, students equipped with frames of paradoxical thinking can rearrange pertinent elements or issues to generate fresh solutions or ideas. Paradoxical thinking also disrupts traditional straight-line reasoning, prompting students to blend conflicting elements or issues into new ideas and solutions. Research provides solid evidence to substantiate this claim. Individuals with paradoxical frameworks demonstrate greater creativity compared to those with alternative cognitive frameworks [56, 57]. Ingram et al. [23] discovered a notably positive impact of paradoxical thinking on innovative behavior. Similarly, later research has indicated that paradoxical mindsets have favorable effects on individual job performance and innovative behavior [26, 62]. Consequently, the following hypothesis was developed:

#### H2

Paradoxical thinking has a substantial effect on the creativity of engineering university students.

### Paradoxical tension and creativity

Paradoxical tensions and responses have the potential to propel learning and innovation, enhance adaptability, and unleash individuals’ potential [29]. Initial studies perceived tension merely as an indicator of anxiety, concentrating on how participants effectively navigate choices between conflicting elements under specific conditions [63]. In contrast to the dilemma perspective that separates conflicting elements, the paradox perspective highlights the competitive yet interconnected associations within tension. Activating the “both-and” mindset in this situation helps individuals discover the positive possibilities of tension over an extended period [61]. Nevertheless, the impact of tension on creative performance remains ambiguous [14, 22, 64].

Moreover, existing studies in this context have predominantly concentrated on the business organization environment [14, 65, 66], with limited investigations in the realm of higher education. The impact of paradoxical tensions on the creativity and innovative behavior of university students is not well-explored, and there is a scarcity of empirical studies examining their experiences with tensions and the resulting implications. Despite existing literature exploring the correlation between paradoxical tensions and innovation (e.g., Ingram et al. [23]; Miron-Spektor et al. [26]), the underlying mechanisms through which paradoxical tensions influence the creativity of engineering university students remain undisclosed. Therefore, the subsequent hypothesis was framed:

#### H3

Paradoxical tensions exert a significant influence on the creativity of engineering university students.

### Mediating role of paradoxical thinking

Personal encounters with paradoxical tensions reflect individuals’ emotional responses, subsequently influencing the frames and processing of their paradoxical cognition, thus impacting their creativity [22, 26, 42, 56, 67]. In simpler terms, when facing tension, students can prompt paradoxical cognition and conscious reasoning, allowing them to establish a paradoxical mindset and cognitive approach [29], ultimately fueling their creativity. Hence, these studies implicitly suggest that paradoxical thinking can mediate the link between experiencing tension and the creativity of university students. Wilson and colleagues demonstrated that teams have the potential to enhance innovative ideas by effectively navigating and blending opposing tensions [68]. Moreover, how university students respond to paradoxical tensions significantly influences whether the tension impedes or enhances creativity, as evidenced by studies conducted by Ingram et al. [23], Jay [24], and Smith & Tushman [29].

Paradoxical thinking mirrors the cognitive frameworks and cognitive activities of university students, along with their cognitive reactions to paradoxical tensions [29, 42]. When students actively welcome and acknowledge enduring paradoxical tensions during the meaning-making process [29, 41], they engage in paradoxical thinking. Consequently, paradoxical thinking can reconfigure and surpass the borders of conflicting matters [45, 53, 60], stimulating the creativity of university students [23, 26, 42]. Hence, the subsequent hypothesis was formulated:

#### H4

Paradoxical thinking plays a mediating role in the relationship between paradoxical tension and the creativity of engineering university students.

### Moderating role of team psychological capital

Tension can lead to negative outcomes, perpetuating a cycle of paradoxical tensions [42]. Team members’ ability to persist despite tension from setbacks will determine their overall success or failure. Team psychological capital promotes a proactive approach to dealing with these paradoxical tensions, fostering active coping and paradoxical thinking [42]. The potential of paradoxical tensions and responses to stimulate intellectual growth and inventive progress, improve adjustability, and tap into human capabilities is evident [42]. Paradoxical thinking empowers engineering university students to actively embrace tensions and wrestle with contradictory yet interconnected issues [42]. Team psychological capital helps alleviate the adverse effects of members’ frustration [69]. Integrating paradoxical elements is crucial in addressing the inherent tensions in innovative efforts within engineering project research and development. Accordingly, the subsequent hypotheses were formulated:

#### H5

Team psychological capital plays a moderating role in the link between paradoxical tension and the creativity of engineering university students.

#### H6

Team psychological capital plays a moderating role in the link between paradoxical tension and paradoxical thinking.

**Fig. 1:**
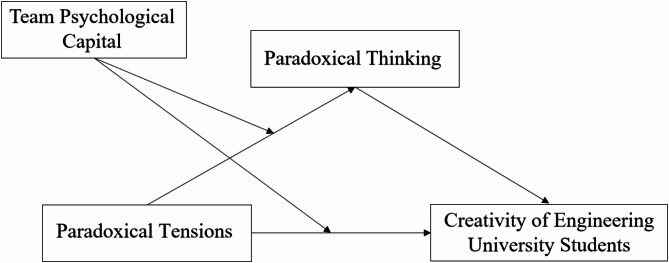
Hypothesized research framework

Figure 1 illustrates the research framework diagram.

## Methods

### PLS-SEM

Considering the complex and multifaceted nature of this study, which includes both moderated and mediated models, PLS-SEM was chosen for analysis. Smart PLS, version 4.0.9.5, was employed to carry out the PLS-SEM analysis. The process followed a two-step approach, evaluating both the measurement and structural models. Rigorous assessments were conducted on the reliability and validity of the measurement model, as well as the explanatory power of the structural model [70].

### Instrumentation

The measurement scales utilized in this research were derived from established literature, validated, and previously employed in similar studies. Additionally, certain items were adjusted to align with our research context (see Appendix A). All items underwent evaluation utilizing a five-point Likert-type scale (1 = “Strongly disagree”, 5 = “Strongly disagree”). Before the main survey, a pilot test involving 150 participants was conducted to minimize potential ambiguity in survey items and integrate necessary modifications that capture creativity among engineering university students and its influencing factors. The survey instrument comprised two primary sections. The first section collected demographic data of the participants (gender, academic major, class level) utilizing fixed nominal scales. The second section encompassed four latent constructs, incorporating 14 indicator items to measure the influencing factors impacting the creativity of engineering university students. The scale was developed based on previous studies [71, 72].

The focus of this research is on the creativity of engineering students, which is considered a general concept rather than a reference to specific individual creative personalities, creative products, creative processes, or creative environments. Therefore, we utilize a scale developed by Zhou & George (2001) to assess the creativity of engineering students [73]. Toh & Miller (2019) and Heininger (2019) have similarly employed this method for measuring creativity [74, 75]. Creativity was measured by four items adapted from [76–77, 72]. We have included the following sample item: “Comes up with different methods to reach goals or objectives and make quality better.” The Cronbach’s alpha, composite reliability (CR), and average variance extracted (AVE) for the scale were 0.937, 0.938, and 0.841, respectively.

Paradoxical tension was evaluated utilizing the three-item scale adapted from Miron-Spektor et al. [26], Bengtsson et al. [78], and Wang & Liu [27]. For instance, in “Paradoxical tension,” we have included the following sample item: “Frequently managing competing demands and addressing them simultaneously.” The Cronbach’s alpha, CR, and AVE for the scale were 0.837, 0.866, and 0.761, respectively.

Paradoxical thinking was measured based on a three-item scale adapted from Miron-Spektor et al. [26] and Ingram et al. [23]. We have included the following sample item: “Gaining a better understanding of an issue through considering conflicting perspectives and accepting contradictions.” The Cronbach’s alpha, CR, and AVE for the scale were 0.943, 0.944, and 0.898, respectively.

Team psychological capital was evaluated using a four-item scale adapted from Avey et al. [79] and Luthans et al. [80]. We have included the following sample item: “I am confident in presenting information to a group of classmates and overcoming difficulties.” The Cronbach’s alpha, CR, and AVE for the scale were 0.929, 0.930, and 0.826, respectively.

To avoid redundancy and intercorrelation among indicators, the validity of the questionnaire was analyzed, focusing on examining and refining the questionnaire measures to ensure its operational feasibility. The study employed the Kaiser-Meyer-Olkin (KMO) measure and Bartlett’s test in SPSS 24.0 to measure the validity of the questionnaire. A KMO above 0.7 and a significance level of *P* < 0.05 in Bartlett’s test are indicative of high questionnaire validity, making it suitable for measuring the respective variables. In this study, the KMO was 0.857 (> 0.7), and the *P*-value was 0.000 (< 0.05), demonstrating the high validity of the questionnaire. These results affirm the effectiveness of the questionnaire in measuring the factors under investigation.

### Target population and data collection

For the comprehensive data collection, the distribution of a modified online questionnaire via WeChat was facilitated using Wenjuanxing (https://www.wjx.cn/). Participants had the option to opt-out if they perceived that their creativity might be adversely affected by participation. Following the “10 times” rule, a minimum sample size of 140 is recommended for models with a maximum of 14 constructs [81]. The questionnaire surveys were distributed to students across eight universities during the spring semester, from June 5 to July 30, 2023. The self-administered surveys were distributed to students in the following universities, along with the corresponding number of participants in parentheses: South China University of Technology (141), Dongguan University of Technology (158), Southern University Science and Technology (135), Shenzhen Technology University (121), Guangdong University of Technology (161), Foshan University (198), Wuyi University (132), Guangdong University of Petrochemical Technology (194), and Guangzhou University (236). In total, 1476 questionnaires were distributed. After excluding 189 questionnaires due to numerous missing values, the analysis focused on 1287 usable questionnaires, resulting in a response rate of 87%. The demographic details of the respondents are shared in Table 1.

**Table 1:** Respondents details

Demographic Variable	*N*	%	Demographic Variable	*N*	%
**Gender**			**Academic Major**		
Male	973	66%	Mechanical Engineering Technology	177	12%
Female	503	34%	Robotics and Automation Engineering	168	11%
**Class Level**			Electrical Engineering and Automation	267	18%
1st Year	443	30%	Manufacturing Engineering	189	13%
2nd Year	421	29%	Automotive Engineering	254	17%
3rd Year	345	23%	Measurement and Control Engineering	288	20%
4th Year	267	18%	IoT Systems Engineering	133	9%

## Results

### Evaluation of measurement model

The study initially converted the cleaned data into a “.csv” format using SPSS 24.0 and imported it into Smart PLS (v.4.0.9.5) to assess the effectiveness of the measurement model. The evaluation of the measurement model within the proposed framework aimed to scrutinize the reliability and validity of the variable measures during the primary data collection phase.

Urbach and Ahlemann [82] have recommended threshold values for exploratory research: CA > 0.8, CR > 0.8, AVE > 0.5, indicator weight > 0.1, and factor loadings (greater than 0.7) > cross-loadings. In this study, the factor-loading values exceeded the cross-loading values, indicating discriminant validity among constructs. Detailed information on all measurement model values is shared in Table 2, where CA surpassed 0.8, CR exceeded 0.8 for all constructs, AVE surpassed 0.5 for all constructs, indicator weight exceeded the threshold value of 0.1, and factor loadings were greater than all cross-loadings, satisfying the criterion for discriminant validity. In summary, these values meet al.l the criteria for determining validity and reliability.

**Table 2:** Indicator weight, loadings, CA, CR, and AVE

Construct / Item	Indicator Weight	Factor Loadings / Cross Loadings			
		PTK	CRE	PT	TPC
**Paradoxical thinking (CA = 0.943, CR = 0.944, AVE = 0.898)**					
PTK1	0.354	**0.938**	0.741	0.631	0.683
PTK2	0.361	**0.955**	0.759	0.660	0.682
PTK3	0.340	**0.950**	0.709	0.630	0.648
**Creativity (CA = 0.937**,** CR = 0.938**,** AVE = 0.841)**					
CRE1	0.267	0.676	**0.905**	0.715	0.725
CRE2	0.277	0.729	**0.911**	0.750	0.718
CRE3	0.269	0.713	**0.918**	0.694	0.728
CRE4	0.277	0.734	**0.935**	0.714	0.748
**Paradoxical tension (CA = 0.837**,** CR = 0.866**,** AVE = 0.761)**					
PT1	0.406	0.616	0.734	**0.930**	0.722
PT2	0.419	0.644	0.750	**0.944**	0.731
PT3	0.314	0.500	0.547	**0.726**	0.517
**Team psychological capital (CA = 0.929**,** CR = 0.930**,** AVE = 0.826)**					
TPC1	0.282	0.668	0.734	0.688	**0.938**
TPC2	0.263	0.609	0.698	0.734	**0.887**
TPC3	0.276	0.662	0.708	0.681	**0.932**
TPC4	0.279	0.634	0.750	0.666	**0.875**

Note: The factor loadings are indicated by bold values. PTK = Paradoxical Thinking, CRE = Creativity, PT = Paradoxical Tension, TPC = Team Psychological Capital, CA = Cronbach’s alpha, CR = Composite Reliability, AVE = Average Variance Extracted

Roemer et al. [83] advocate that the Heterotrait-Monotrait ratio (HTMT) proves advantageous in appraising discriminant validity when dealing with measurement models. Our discriminant validity assessment aligns with the criteria set forth by Henseler et al. [84]. The values must remain below 0.900. Fornell and Larcker [85] suggest that the square root of the AVE should be greater than the correlation between the construct and other constructs. This study fulfills this criterion. The findings have been tabulated in Table 3.

**Table 3:** Results of Fornell-Larcker and HTMT analyses

	PTK	CRE	PT	TPC
PTK	**0.948**	0.826	0.760	0.756
CRE	0.777	**0.917**	0.880	0.852
PT	0.676	0.783	**0.872**	0.859
TPC	0.709	0.796	0.761	**0.909**

Note: The lower triangle represents the Pearson correlation coefficients between constructs, while the diagonal contains the square roots of the AVE

The upper triangle represents the Heterotrait-Monotrait ratio (HTMT) analysis

PTK = Paradoxical Thinking, CRE = Creativity, PT = Paradoxical Tension, TPC = Team Psychological Capital, AVE = Average Variance Extracted

### Evaluation of structural model

The R^2^ values, scaling from 0 to 1, are used to gauge predictive accuracy, where 0.750 is substantial, 0.500 is moderate, and 0.250 is weak. In the current research, all values exceeded 0.500 (moderate); creativity exhibited the highest R^2^ (0.762), while paradoxical thinking had the least robust R^2^ (0.549).

The predictive relevance (Q^2^) of the model was determined, with appropriate Q^2^ values considered crucial for accurate predictions of indicator points. A Q^2^ value > 0 indicates successful predictive relevance for the variable (0.020 as small, 0.150 as medium, and 0.350 as large). A blindfolding process within the Smart PLS (v.4.0.9.5) tool was employed to determine Q^2^ values for the dependent variables. In this study, all Q^2^ values for the variables exceeded 0, indicating robust support for the predictive relevance of the model across all dependent variables.

The goodness-of-fit (GOF) assesses predictive model accuracy, with 0.10 indicating a poor fit, 0.25 acceptable, and 0.36 good [81]. In this study, the calculated model fit was 2.021, surpassing 0.36, indicating a generally high overall fit for the model.

### Hypotheses assessment

Table 4 presents the findings from hypotheses testing. The first hypothesis suggests a positive connection between paradoxical tension and paradoxical thinking. The outcomes suggested a positive association (t = 14.098, *P* < 0.001) between paradoxical tension and paradoxical thinking, providing support for H1. Effect sizes (f^2^) gauge the magnitude of a variable’s influence on the dependent variable. The recommended benchmarks for interpretation are as follows: 0.02 to 0.15 (small), 0.15 to 0.35 (medium), and > 0.35 (large) [86]. The actual impact of paradoxical tension on paradoxical thinking is considered small (f^2^ = 0.099).

The second hypothesis suggests a positive relationship between paradoxical thinking and the creativity of engineering university students. The outcomes suggested a positive association (t = 4.536, *P* < 0.001) between paradoxical thinking and creativity, providing support for H2. The actual impact of paradoxical thinking on creativity is considered medium (f^2^ = 0.224).

The third hypothesis posits a positive connection between creativity in engineering university students and paradoxical tension. The findings revealed a significant positive association (t = 11.861, *P* < 0.001) between paradoxical tension and creativity, supporting H3. The observed impact of paradoxical tension on creativity is assessed as medium (f^2^ = 0.154).

The fourth hypothesis suggests that paradoxical thinking mediates the relationship between paradoxical tension and creativity in engineering university students., enhancing the association between them. The findings indicated that the impact of paradoxical tension on creativity is channeled through the mediating effects of paradoxical thinking, thereby supporting the hypothesis.

The fifth hypothesis suggests that team psychological capital, within the context of engineering university students, assumes a moderating role between paradoxical tension and creativity. The outcomes revealed that moderating effects of team psychological capital amplify the influence of paradoxical tension on creativity, providing support for H5. The real impact of team psychological capital on paradoxical tension and creativity falls within the medium range (f^2^ = 0.146).

The sixth hypothesis posits that the moderating influence of team psychological capital will enhance the impact of paradoxical tension on paradoxical thinking. However, the findings indicated that team psychological capital does not amplify the effect of paradoxical tension on paradoxical thinking, thereby not supporting H6. The impact of team psychological capital on both paradoxical tension and paradoxical thinking is of medium magnitude (f^2^ = 0.181).

**Table 4:** Hypotheses assessment

Hypothesis	Original Sample (O)		Standard Deviation (STDEV)	T Statistics (t-value)	Confidence Intervals 2.5%: 97.5%	Effect Size (f^2^)	Support
Total Effects							
H1: PT → PTK		0.634	0.045	14.098***	0.551, 0.724	0.099^small^	YES
H2: PTK → CRE		0.236	0.052	4.536***	0.151, 0.349	0.224^medium^	YES
H3: PT → CRE		0.572	0.048	11.861***	0.473, 0.660	0.154^medium^	YES
Mediation Effect of Paradoxical Thinking							
H4: PT → PTK → CRE		0.150	0.036	4.101***	0.090, 0.230		YES
Moderation Effect of Team Psychological Capital							
H5: TPC × (PT → CRE)		-0.069	0.009	7.263***	-0.088, -0.051	0.146^medium^	YES
H6: TPC × (PT → PTK)		-0.016	0.008	2.025	-0.032, -0.001	0.181^medium^	NO

Note: T > 1.96 at *P* < 0.05 (*), T > 2.576 at *P* < 0.01 (**), and T > 3.29 at *P* < 0.001 (***)

PTK = Paradoxical Thinking, CRE = Creativity, PT = Paradoxical Tension, TPC = Team Psychological Capital

## Discussion and conclusion

### Discussion

#### Regarding Question 1: what is the impact of paradoxical tensions and paradoxical thinking on the creativity of engineering students?

This research explores the correlation between paradoxical tension, paradoxical thinking, and the creativity of university engineering students. Additionally, the study examines the moderating effect of team psychological capital in this context. The results reveal that both paradoxical tension and paradoxical thinking significantly impact the creativity of engineering university students. Furthermore, paradoxical tension has a significant influence on paradoxical thinking. Simultaneously, paradoxical tension impacts creativity through the mediation of paradoxical thinking, indicating a significant mediating role of paradoxical thinking. Team psychological capital emerges as a significant moderator between paradoxical tension and creativity, while its moderating effect is not significant in the link between paradoxical tension and paradoxical thinking.

#### The relationship between paradoxical tension and paradoxical thinking

The observed significant influence of paradoxical tension on paradoxical thinking aligns with the findings of Smith et al. [87] and Wang & Liu [27]. The creative process in engineering necessitates a careful balance between seemingly opposing factors. This equilibrium arises from the tension created by conflicting design goals, resource limitations, and shifts in technological approaches, serving as a crucial driver for inventive issue resolution. This study posits that deliberately cultivating a state of paradoxical tension among engineering students can serve as a catalyst, inspiring engineers to skillfully address and harmonize conflicting requirements, ultimately nurturing inventive solutions.

#### The relationship between paradoxical thinking and creativity of engineering university students

The significant impact of paradoxical thinking on the creativity of engineering university students aligns with the outcomes of Yang et al. [88] and Litchfield et al. [89]. Paradoxical thinking entails the ability to embrace contradictory elements, creating a unique cognitive framework that encourages adaptability and innovative ideation. This outcome underscores the potentially transformative impact of incorporating paradoxical thinking into teaching methods within engineering programs. Amidst an era shaped by complex technological challenges, developing thinking that adeptly handles uncertainty and intricacy becomes crucial. Recognizing and appreciating the contradictions inherent in engineering problems not only expands the intellectual perspectives of the students but also cultivates resilience essential for tackling diverse real-world challenges.

#### The relationship between paradoxical tension and creativity of engineering university students

The significant impact of paradoxical tension on the creativity of engineering university students aligns with the findings of Miron-Spektor et al. [90] and Gaim & Wåhlin [91]. Exploring how paradoxical tension affects creativity in engineering students delves deep into the heart of thinking processes, challenging traditional ways of thinking. Paradoxical tension, with its conflicting elements, acts as a mental crucible that promotes transformative thinking. It compels students to navigate contradictions, resulting in innovative solutions beyond conventional methods. Embracing this tension as essential to engineering creativity redefines education, highlighting the importance of an environment that nurtures conflicting thoughts. Acknowledging and harnessing this creative potential enables students to address complex challenges, preparing them as flexible and forward-thinking engineers in a rapidly evolving technological landscape.

#### Regarding Question 2: what role does paradoxical thinking play in the correlation between paradoxical tensions and creativity in engineering students?

The pivotal role of paradoxical thinking in linking paradoxical tension with engineering creativity is evident, a conclusion supported by Ingram et al. [23] and Jay [24]. The combination of paradoxical tension and engineering creativity creates a back-and-forth interaction where seemingly conflicting elements converge, fostering an environment conducive to transformative insights. This interdependent relationship suggests that paradoxical thinking acts as the crucible wherein tensions inherent in engineering challenges are not only recognized but actively used to drive creative thinking. The essence of paradoxical thinking lies in its ability to navigate the delicate balance between opposing forces, catalyzing the blending of diverse perspectives. It emerges as the key cognitive element that resolves apparent contradictions in engineering pursuits, surpassing traditional thought boundaries. Paradoxical thinking infuses the creative process with increased resilience through its mediation, enabling engineers to move beyond binary oppositions and embrace the collaborative potential within paradoxical tensions.

#### Regarding Question 3: what role does team psychological capital play in positively or negatively moderating the influence between paradoxical tensions and paradoxical thinking, and between paradoxical tensions and creativity?

The impact of team psychological capital is crucial when examining the moderation between paradoxical tension and creativity. It does not exert a considerable moderating influence on the link between paradoxical tension and paradoxical thinking. The substantial impact of team psychological capital on the connection between paradoxical tension and creativity implies that psychological resources at the team level are vital in utilizing the positive aspects of paradoxical tension within a team setting. This underscores the capacity of the team to use collectively shared positive psychological resources to manage and capitalize on the inherent tensions in paradoxical situations, ultimately creating a favorable environment for creative results. However, the non-significant moderating effect between team psychological capital and paradoxical thinking implies that, while team-level positive psychological capital may facilitate the creative potential arising from tension, its impact may not extend to the cognitive strategies associated with navigating paradoxical thinking at the team level.

## Conclusion

### Theoretical contributions

The observed interaction between paradoxical tension and paradoxical thinking in engineering creativity constitutes substantial theoretical contributions, enhancing our understanding of the complex mechanisms at play. These insights advance theoretical ideas and offer a conceptual perspective. Theoretical advancements in this domain contribute to a deeper understanding of how students can be trained to approach design challenges with a mindset that embraces and synthesizes paradoxes, ultimately yielding innovative and original solutions.

Team Psychological Capital significantly moderates the relationship between Paradoxical Tension and Creativity. This discovery adds to theoretical complexity by highlighting the significance of team-level psychological resources as crucial components in magnifying or diminishing the impact of tension caused by paradoxes on creative results. The integration of team psychological capital into the theoretical discourse not only enhances our understanding of paradoxical dynamics but also stimulates additional investigation of team-oriented psychological factors in the broader scope of creative processes.

### Practical implications

The significant impact of paradoxical tension and paradoxical thinking on engineering creativity underscores the importance of embracing conflicting thoughts when addressing problems in engineering situations. The considerable influence of paradoxical tension, coupled with paradoxical thinking, highlights the necessity for engineering teams to adopt a mindset that values complexity and contradiction as catalysts for creative solutions. Embracing conflicting thoughts and accommodating contradictions is not merely a theoretical concept but a crucial practical approach with tangible benefits for innovation and problem-solving.

Additionally, the role played by team psychological capital in the connection between paradoxical tension and creativity underscores the importance of cultivating a positive team psychological atmosphere. In practical terms, these findings imply that organizations in the engineering field should not only support the development of paradoxical thinking but also invest resources into strategies for establishing and maintaining positive team psychological capital. Recognizing and addressing paradoxes in engineering projects serve as a practical approach to stimulate creative problem-solving. This emphasizes the necessity for leadership to foster a culture that values cognitive diversity, embraces contradictions, and invests in the psychological well-being of engineering teams to optimize their creative potential in tackling complex challenges.

### Limitations

This research has some limitations that might act as catalysts for further exploration of the subject. First, although our study employed a cross-sectional design with a three-month gap between data collection points, we acknowledge the limitations in drawing definitive causal inferences from our mediation analysis and suggest that future research employing longitudinal methods, such as experience sampling, could provide a more nuanced understanding of potential changes in work-related tensions over time. Second, the participants in this study are exclusively from China, suggesting the importance of subsequent investigations that involve cross-cultural comparisons to discern the potential impact of paradoxical tension and paradoxical thinking on the engineering creativity of students across diverse geographical regions. Third, the data were collected through self-report measures, introducing the possibility of common method bias to the results. While students may possess insight into their feelings and choices, future studies should consider using alternative data sources, such as teachers or administrators, to obtain more objective data. Fourth, we have primarily investigated the relationship between paradoxical thinking and creativity and have not included other types of thinking that may influence the creativity of engineering students, such as unconscious thinking and design thinking, in our discussion. Future research could explore the impact of other thinking models on creativity. Finally, while our study operationalizes “creativity” through a measure that closely aligns with creative self-belief, we acknowledge that creativity is a complex concept. By focusing on a specific aspect of it, our study offers greater practical relevance. We recognize this as a limitation and suggest that our findings are particularly pertinent to the domain of self-perceived creativity. Future research could explore creativity from a broader perspective.

## Electronic supplementary material

Below is the link to the electronic supplementary material.

Supplementary Material 1

## Data Availability

The raw data supporting this study’s conclusions will be made available by the authors (dora_guo@e.gzhu.edu.cn or dora777guo@gmail.com) without undue reservation.

## Footnotes

1 Zhong-Yong thinking, as a cognitive style, is characterised by a comprehensive consideration of issues from multiple perspectives, a circumspect avoidance of extremism, a propensity for contextually appropriate conduct, and a commitment to the preservation of harmony [92, 93]. Chinese students, influenced by the Zhong-Yong culture, are adept at managing paradoxical thinking and tensions due to their training in considering multiple perspectives, avoiding extremes, and seeking harmonious solutions, which collectively enhance their creative capabilities [94, 95].

2 The Yin-Yang concept recognises the coexistence of opposing, yet interconnected elements that are both contradictory and complementary, existing simultaneously and synergistically [96–99]. Yin-Yang philosophy indicates that seemingly contradictory forces are not only inevitable but also necessary for balance and harmony [100, 101]. This understanding allows Chinese students to embrace and navigate paradoxes more comfortably, as they are accustomed to thinking in terms of interrelated and complementary opposites, rather than viewing situations as strictly binary or mutually exclusive [44, 50].

## Abbreviations

AVE: Average Variance Extracted
CA: Cronbach’s alpha
CR: Composite Reliability
CRE: Creativity
PT: Paradoxical Tension
PTK: Paradoxical Thinking
TPC: Team Psychological Capital
