# Supplementary material for: Exploring the roles of paradoxical tensions, paradoxical thinking, and team psychological capital on the creativity of engineering university students
Source: BMC Psychol. 2025 Feb 12;13:117. doi: 10.1186/s40359-025-02427-3 (PMC11823069; doi:10.1186/s40359-025-02427-3)
Supplement: Supplementary file 1 — Supplementary Material 1 [file 40359_2025_2427_MOESM1_ESM.docx]

**APPENDIX A**

**Supplementary material: Survey questionnaire**

Dear Participants,

Thank you for taking the time to participate in our survey. We are conducting a study to explore the relationship between paradoxical tensions, paradoxical thinking, team psychological capital, and the creativity of engineering students. Your responses are confidential and will be used for research only.

Thank you for your participation.

**DEMOGRAPHIC QUESTIONS**

**What is your gender?**

- Male 🞎
- Female 🞎

**What is your Class Level?**

- 1st Year 🞎
- 2nd Year 🞎
- 3rd Year 🞎
- 4th Year 🞎

**What is your current Academic Major? （Fill in your answers in the blanks）**

- __________________________________________________________

**SCALE SECTION**

Please select the rating that best describes your experience, where 1 means “Strongly agree”, 2 means “Agree”, 3 means “Neutral”, 4 means “Disagree”, and 5 means “Strongly disagree”.

| Variable | Measurement Items | (1) | (2) | (3) | (4) | (5) |
| --- | --- | --- | --- | --- | --- | --- |
| Creativity (CRE) | | | | | | |
| CRE1 | Comes up with different methods to reach goals or objectives and make quality better. |  |  |  |  |  |
| CRE2 | Look for new and useful technologies, processes, techniques, and/or product ideas to make performance better. |  |  |  |  |  |
| CRE3 | Has a lot of creative and innovative ideas. |  |  |  |  |  |
| CRE4 | Do not fear taking risks and giving creative solutions to problems. |  |  |  |  |  |
| Paradoxical Tension (PT) | | | | | | |
| PT1 | Frequently managing competing demands and addressing them simultaneously. |  |  |  |  |  |
| PT2 | Holding contradictory ideas and frequently encountering conflicting goals. |  |  |  |  |  |
| PT3 | Regularly dealing with contradictory requirements, often needing to decide between opposing alternatives. |  |  |  |  |  |
| Paradoxical Thinking (PTK) | | | | | | |
| PTK1 | Gaining a better understanding of an issue through considering conflicting perspectives and accepting contradictions. |  |  |  |  |  |
| PTK2 | Enjoying the pursuit of contradictory goals and feeling energized when addressing contradictory issues. |  |  |  |  |  |
| PTK3 | Frequently experiencing oneself as simultaneously embracing conflicting demands. |  |  |  |  |  |
| Team Psychological Capital (TPC) | | | | | | |
| TPC1 | I am confident in presenting information to a group of classmates and overcoming difficulties. |  |  |  |  |  |
| TPC2 | If I should find myself in a jam trying to solve this situation, I could think of many ways to get out of it. |  |  |  |  |  |
| TPC3 | I take stressful things regarding setbacks in stride. |  |  |  |  |  |
| TPC4 | I look on the bright side of things regarding challenges. |  |  |  |  |  |
